# Epidemiology of Alpine Skiing and Snowboarding Injuries: A Systematic Review and Meta‐Analysis

**DOI:** 10.1002/hsr2.73252

**Published:** 2026-09-13

**Authors:** Juho Laaksonen, Matias Vaajala, Rasmus Liukkonen, Oskari Pakarinen, Ville M. Mattila, Ilari Kuitunen

**Affiliations:** ^1^ Department of Clinical Medicine University of Tampere Tampere Finland; ^2^ Department of Surgery Central Finland Central Hospital Jyväskylä Finland; ^3^ Department of Surgery Päijät‐Häme Central Hospital Lahti Finland; ^4^ Department of Orthopedics and Traumatology Tampere University Hospital Tampere Finland; ^5^ Coxa Hospital for Joint Replacement Tampere Finland; ^6^ Institute of Clinical Medicine and Department of Pediatrics University of Eastern Finland Kuopio Finland; ^7^ Department of Pediatrics Kuopio University Hospital Kuopio Finland

**Keywords:** alpine skiing, incidence, injury, snowboarding

## Abstract

**Objectives:**

To investigate the incidence of injuries in alpine skiing and snowboarding, while identifying common injury locations and types.

**Methods:**

A systematic review and meta‐analysis were conducted by searching PubMed, Web of Science, CINAHL and Scopus in March 2025 (Prospero database CRD420251010125). Studies reporting injury incidence in alpine sports were included. Pooled injury incidence rates were calculated using a random‐effects model, with analyzes stratified by participation level, sport, anatomical location, and injury type.

**Results:**

A total of 82 studies were included. Ten studies at the professional level reported 2165 injuries over 21,806,100 runs, corresponding to an overall injury incidence of 0.01 per 1000 runs (95% CI: 0.00–0.03). Professional alpine skiing had an incidence of 0.02 per 1000 runs (95% CI: 0.01–0.06) whereas professional snowboarding showed injury rate of 3.8 per 1000 runs (95% CI: 1.2–12.2). At the amateur level, 43 studies documented a total of 424,517 injuries over 408,624,850 skier‐days. This corresponds to a pooled injury incidence of 1.9 injuries per 1000 skier‐days (95% CI: 1.3–2.7). Amateur alpine skiing had an injury incidence of 3.8 per 1000 skier‐days (95% CI: 2.3–6.1), whereas amateur snowboarding had 1.4 per 1000 skier‐days (95% CI: 0.1–27.0). Limb injuries were the most common in both groups, while trunk injuries were additionally frequent among professionals. Among amateurs, fractures and sprains were the most frequently reported injury types, and falls were the leading mechanism of injury. However, substantial between‐study heterogeneity (I^2^ > 99% for most analyzes) should be considered when interpreting the pooled estimates.

**Conclusion:**

Professional snowboarders experienced higher injury incidence than alpine skiers, whereas the opposite trend was observed among amateurs. Limb injuries were the most common in both groups, while trunk injuries were more frequent among professionals.

## Summary

**What is Already Known on This Topic**
  - Much of the literature on injury incidence in alpine skiing and snowboarding is outdated, with many studies published in or before the early 2000s.
  - Existing reviews are largely narrative and often do not provide pooled injury incidence estimates.
  - Previous research has predominantly focused on professional athletes, with limited data on amateur participants.
**What This Study Adds**
  - Injury prevention strategies should be tailored to both skill level and sport, rather than using a single approach for all alpine snow sports.
  - Injury incidence was higher in professional snowboarding than alpine skiing, whereas amateurs showed higher rates in alpine skiing.
  - Limb injuries predominated in both groups, with trunk injuries more common among professionals.
  - Differences in exposure metrics prevented direct comparisons between amateur and professional populations.
**How This Study Might Affect Research, Practice or Policy**
  - Injury prevention strategies should be tailored to both skill level and sport, rather than using a single approach for all alpine snow sports.
  - Among professionals, prevention efforts may focus on trunk protection, collision avoidance, and safe landing techniques, while amateur interventions should emphasize fall education, equipment adjustment, and terrain awareness.
  - The prominence of falls among amateurs suggests that slope design, hazard management, and safety policies may contribute to injury reduction.

## 1 Introduction

Alpine skiing and snowboarding are widely practiced recreational and competitive sports that attract millions of participants worldwide each year [1]. Although these sports provide substantial physical and psychological benefits [2], they are also associated with a considerable risk of injury because of high speeds, challenging environmental conditions, and the potential for falls and collisions. Injuries range from minor sprains and contusions to severe fractures, concussions, and spinal trauma, which may result in prolonged disability or premature retirement from sport [3, 4]. Injury incidence and patterns may differ according to discipline and level of participation, with factors such as equipment and skill level influencing injury risk [5].

Over the past two decades, alpine snow sports have undergone notable evolution in both recreational and professional settings. Technological advancements in equipment, such as shaped skis, rocker profiles, and enhanced snowboard bindings, have changed how athletes interact with the snow, potentially influencing biomechanical loads and injury risk. Competition formats have become faster and more technically complex, while the widespread use of terrain parks has introduced new injury mechanisms, particularly among younger and amateur participants [6, 7]. The introduction of carving skis in the late 1990s and early 2000s represented the most significant equipment innovation in alpine skiing, fundamentally changing turning mechanics and performance demands [8]. Moreover, the increased adoption of safety gear, such as helmets and back protectors, may have altered both the type and severity of injuries [9, 10].

Despite these developments, much of the existing literature on injury incidence in alpine sports is outdated, with a large proportion of studies published in or before the early 2000s [11]. In addition, previous reviews have either been narrative in nature, often lacking pooled injury incidence estimates, or have focused exclusively on professional‐level athletes [12, 13, 14]. As alpine skiing and snowboarding continue to evolve in terms of equipment, participation patterns, and competition formats, up‐to‐date and comprehensive epidemiological data are essential for accurately understanding current injury trends and guiding effective injury‐prevention strategies.

The aim of this systematic review and meta‐analysis was to assess and compare injury incidence in alpine skiing and snowboarding, stratified by level of participation (amateur vs. professional) and discipline. Secondary outcomes included the distribution of injuries according to anatomical location, injury type, and injury mechanism. In addition, a post hoc time‐stratified analysis comparing studies published before and after 2000 was performed to explore potential changes in injury patterns associated with the introduction of modern ski equipment. By synthesizing the existing evidence, this review seeks to inform the development of targeted, evidence‐based injury‐prevention strategies and to enhance safety across all levels of alpine snow sports.

## 2 Methods

### 2.1 Search Process

In March 2025, a systematic search was conducted in the PubMed (National Library of Medicine), Web of Science (Clarivate), Scopus (Elsevier), and CINAHL (EBSCO) databases using the search terms: “(alpine skiing OR slalom OR skiing OR snowboarding) AND (injury OR fracture).” Full database‐specific search strategies are provided in the Supporting Information S4 (SearchAlgorithm_alpine). The search differed from the original protocol, as this review also included snowboarding in addition to alpine skiing. This protocol deviation was made at the beginning of the study during the search phase to broaden the scope of the review and include both major alpine snow sports. A second deviation occurred during the analysis phase, when a post hoc stratification of injuries by studies published before versus after 2000 was introduced to explore potential changes in injury patterns following the introduction of carving skis. The search results were imported into Covidence software (Veritas Healthcare Inc, Melbourne, Victoria), where duplicate entries were automatically removed. Five authors participated in study screening process and all studies were screened independently by two authors, resolving any disagreements through consultation with a third reviewer.

### 2.2 Inclusion and Exclusion Criteria

Only original studies published in peer‐reviewed journals, including both observational and interventional designs, that reported injury incidence with exposure time or exposure units in alpine skiing or snowboarding were considered for inclusion. Studies reporting general skiing without specifying alpine disciplines were excluded. Athletes of all ages, sexes, and skill levels, whether amateurs or professionals, were included, with no exclusions based on injury definitions. Injury definitions were accepted as reported by the original studies and were not used as an eligibility criterion. Studies employing medical‐attention, time‐loss, self‐reported, clinical diagnosis, or combined injury definitions were therefore included. Because approximately 85% of the included studies used medical‐attention injury definitions, whereas the remaining definition categories each comprised only a small number of studies, sensitivity analyzes restricted to individual injury definition categories were considered but were not performed due to insufficient subgroup sizes and limited interpretability. The variability in injury definitions was recognized as a potential source of between‐study heterogeneity and is considered when interpreting the findings. Studies lacking original data, such as reviews, editorials, and commentaries, were excluded, as well as studies not published in English or those with inaccessible full texts.

### 2.3 Data Extraction

Five reviewers participated in the screening process. Titles and abstracts were independently assessed in pairs, followed by full‐text evaluation of all eligible reports. Any disagreements were resolved through discussion, and when needed, by consultation with a third reviewer to minimize potential extraction errors. Data were extracted from the text or figures of the original reports. No automated or semi‐automated tools were used for screening; all title–abstract and full‐text records were reviewed manually by independent reviewers. In cases of suspected duplicate reporting (e.g., similar authors, study periods), we selected the most comprehensive or most recent report to minimize population overlap. Authors of primary studies were not contacted to clarify eligibility for inclusion in this review and reference lists of original studies were not screened. The extracted information included study details: authors, study title, year of publication, country, study duration, and design. Participant characteristics, including competition level (professional or amateur), activity type (training or racing), age group (as categorized by the original study), and sex distribution, were recorded. Additionally, the number of participants, injury definitions, and whether injuries occurred during training or competition were documented.

Key data points included the total number of reported injuries, exposure time, and the units used for measuring exposure. If either total exposure or injury count was missing, they were derived from the incidence rate when provided by the original authors. Additionally, injuries were systematically categorized based on anatomical location (head, trunk, lower limb, upper limb) and type (concussion, strain, sprain, fracture, or other injuries such as lacerations) following the classification system of the Olympic Committee [15] to ensure standardized data collection and analysis. The complete extracted dataset is provided in the Supporting Information S1 (Alpine_data. xlsx).

### 2.4 Outcome Measures

The primary outcome was the overall injury incidence in alpine sports, stratified by level of participation (amateur vs. professional) and by discipline (alpine skiing and snowboarding). Injury incidence was expressed as the number of injuries per 1000 skier/snowboarding‐days or runs depending on study. Exposure was quantified using skier‐days (one individual skiing for any part of 1 day) and runs (one completed descent). For each study, this was calculated by dividing the total number of injuries by the total number of skier‐days/runs and multiplying the result by 1000. Because no validated conversion factors exist between skier‐days, skier runs, skier seasons and exposure hours, exposure metrics were not converted into a common denominator. Instead, studies reporting the same exposure metric were pooled separately, while studies using incomparable exposure metrics were synthesized narratively according to SWiM [16]. Secondary outcomes included the distribution of injuries by anatomical location and injury type. In addition, we performed a time‐stratified analysis, separating studies published before the year 2000 from those published thereafter, to account for the introduction of carving skis, which represented a major equipment change in alpine skiing [8]. This stratification was not prespecified in the original protocol but was introduced during the review process to strengthen the analysis and better reflect potential changes in injury patterns over time.

### 2.5 Risk of Bias

The risk of bias was evaluated using the Joanna Briggs Institute Critical Appraisal Checklist for Prevalence Studies [17]. The evaluation covered nine domains, and each domain was rated as “yes,” or “no.” Studies scoring 8–9 were considered low risk of bias, 6–7 moderate risk, and ≤ 5 high risk of bias. Risk of bias was independently assessed by two reviewers, with any disagreements resolved through adjudication by a third reviewer. The risk‐of‐bias assessment was used to describe the methodological quality of the included studies and did not inform study selection or any primary, subgroup, or sensitivity analyzes.

### 2.6 Statistics

Statistical analyzes followed the guidelines outlined in the latest Cochrane Handbook for Systematic Reviews of Interventions [18]. Injury incidence was calculated individually for each included study, and pooled estimates with 95% confidence intervals (CIs) were derived using a random‐effects meta‐analysis of rates (R package meta). Separate meta‐analyzes were performed for each exposure metric. Studies reporting incompatible exposure metrics were not quantitatively pooled. This model was chosen to account for variations across studies, including differences in participant characteristics such as prior injury history and physical performance. Given the inclusion of diverse injury definitions, some heterogeneity in injury types was expected. Study heterogeneity was assessed using the I^2^ statistic. To explore sources of variability, subgroup analyzes were conducted based on injury location, and injury type. Results were presented visually through forest plots, and all statistical analyzes were performed using the meta package in R (version 4.1.2).

### 2.7 Reporting

This systematic review and meta‐analysis followed the guidelines outlined in the Preferred Reporting Items for Systematic Reviews and Meta‐Analyzes (PRISMA) and the PERSIST (Prisma for Exercise, Rehabilitation, Sport Medicine, and Sports Science) framework [19, 20]. PRISMA checklist is provided in the Supporting Information S3 (PRISMA_2020_checklist‐alpine. docx).

### 2.8 Permissions and Registration

This study did not require research permission due to its design. Our protocol was registered in the Prospero database.

## 3 Results

**Figure 1:**
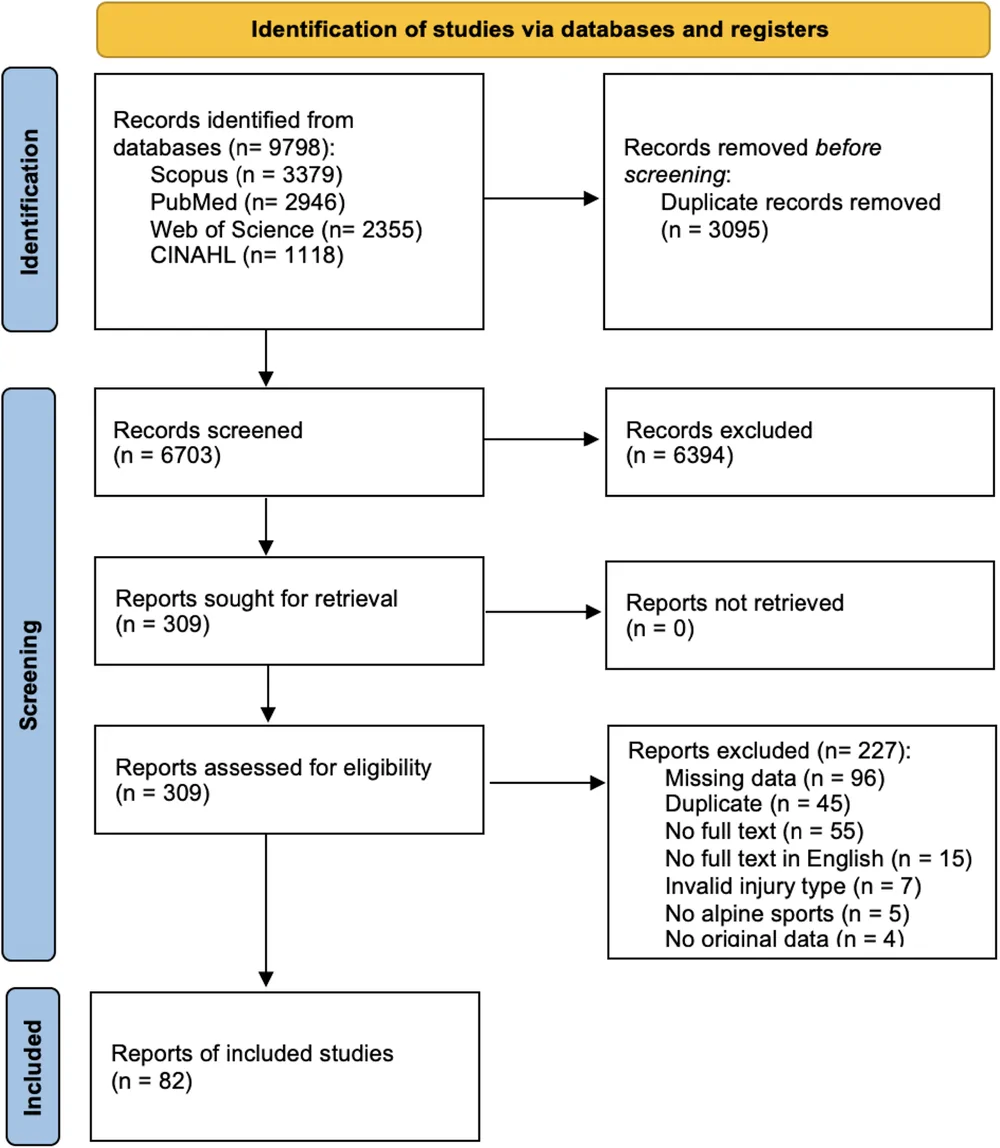
PRISMA flowchart of the study selection process.

A total of 9798 articles were identified through database searches. After removing 3095 duplicates, 6703 articles remained for title and abstract screening. Of these, 309 articles were assessed in full, and 82 studies were ultimately included in the systematic review [21, 22, 23, 24, 25, 26, 27, 28, 29, 30, 31, 32, 33, 34, 35, 36, 37, 38, 39, 40, 41, 42, 43, 44, 45, 46, 47, 48, 49, 50, 51, 52, 53, 54, 55, 56, 57, 58, 59, 60, 61, 62, 63, 64, 65, 66, 67, 68, 69, 70, 71, 72, 73, 74, 75, 76, 77, 78, 79, 80, 81, 82, 83, 84, 85, 86, 87, 88, 89, 90, 91, 92, 93, 94, 95, 96, 97, 98, 99, 100, 101]. The majority of studies were conducted in Europe (*n* = 39, 48%), including 21 from Nordic countries, followed by North America (*n* = 24, 29%), of which 20 were from the United States, and Asia (*n* = 14, 17%), with 12 conducted in Japan. The median year of publication was 2005, the mode was 2001, and the included studies reported data collected between 1978 and 2024. Among the included studies, 33 were retrospective cohort designs, 48 were prospective cohort studies, and one was a randomized controlled trial. One study [94] included both prospective and retrospective analyzes, which were reported separately; therefore, this study was divided into two entries in our analysis. Most studies reported injuries requiring medical attention (any injury that needs a doctor, physiotherapist, or other medical staff, such as ski patrol, to evaluate or treat it) (*n* = 70, 85%). A PRISMA flowchart of the study selection process is provided in Figure 1, and detailed study characteristics are available in Supporting Information Appendix S1.

### 3.1 Risk of Bias

A risk of bias assessment was conducted for all included studies (Supporting Information Appendix S2). Overall, the quality of the included studies was relatively high. Of the 82 studies, 22 (27%) were rated as high quality (with 8 or more criteria properly addressed), while 48 (59%) were classified as moderate quality (6–7 criteria met). Twelve studies (15%) were identified as low quality, scoring 5 or below according to the RoB assessment. The most common issues were incomplete descriptions of the study subjects and settings, as well as the use of non‐standard or unreliable methods to measure the condition. However, most studies employed appropriate statistical analyzes, used sample frames suitable for addressing the target population, and conducted data analysis with sufficient coverage of the identified sample.

### 3.2 Injury Incidences at the Professional Level

**Figure 2:**
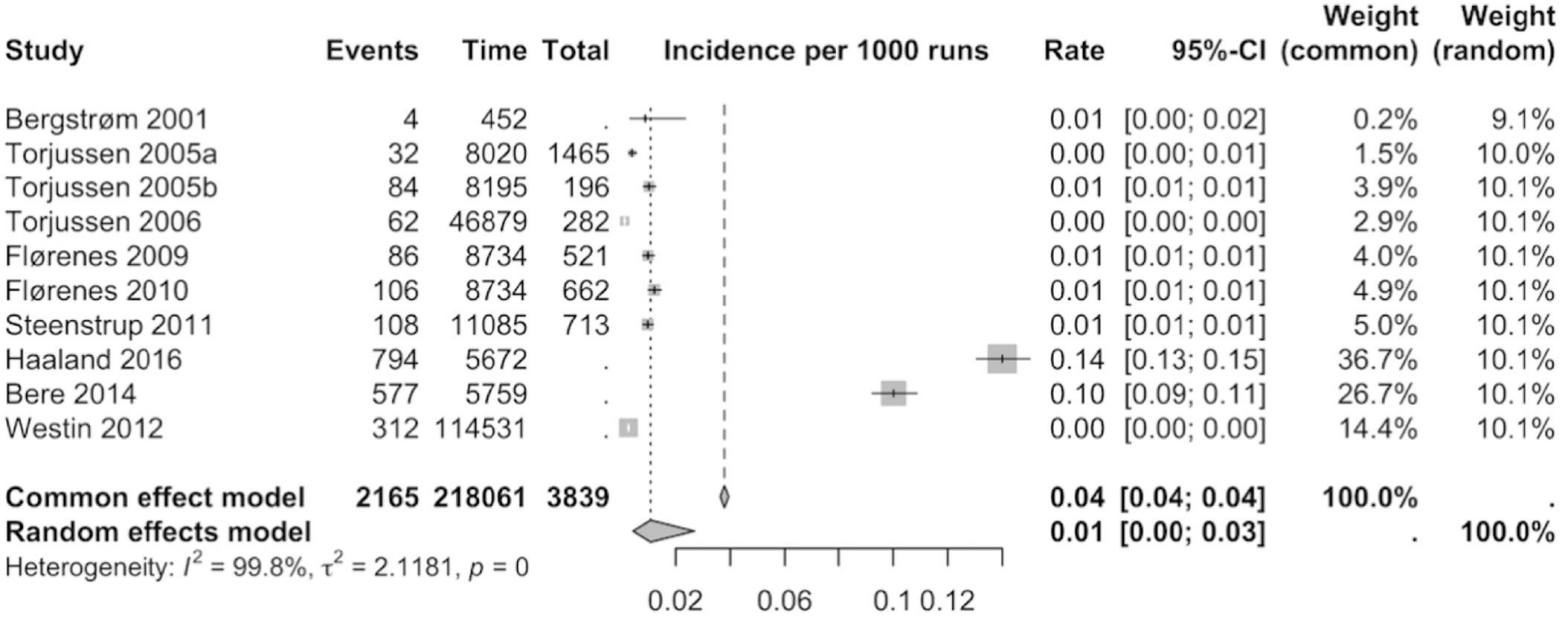
A forest plot illustrating the overall pooled injury incidence per 1000 runs in professional level alpine sports, with exposures represented in thousands.

Injuries at the professional level in alpine snow sports were reported using three different exposure units. Ten studies using runs as the exposure metric reported 2,165 injuries over 218,061,000 runs, corresponding to an injury rate of 0.01 per 1000 runs (95% CI: 0.00–0.03, *I* ^*2*^ = *99.8%, τ^2^=2.1181*; number of studies = 10) (Figure 2). Two studies used skier‐seasons as the exposure unit, reporting 315 injuries across 698 skier‐seasons, with an incidence of 0.6 per 1 skier‐seasons (95% CI: 0.1–2.6, *I* ^*2*^ = *99.5%, τ^2^=1.2019;* number of studies = 2) (Supporting Information Appendix S3). Two additional studies used exposure hours, documenting 98 injuries over 760 h, resulting in an incidence rate of 320.6 per 1000 h (95% CI: 28.4–3616.1, *I* ^*2*^ = *99.2%, τ^2^=3.0322*; number of studies = 2) (Supporting Information Appendix S4).

**Figure 3:**
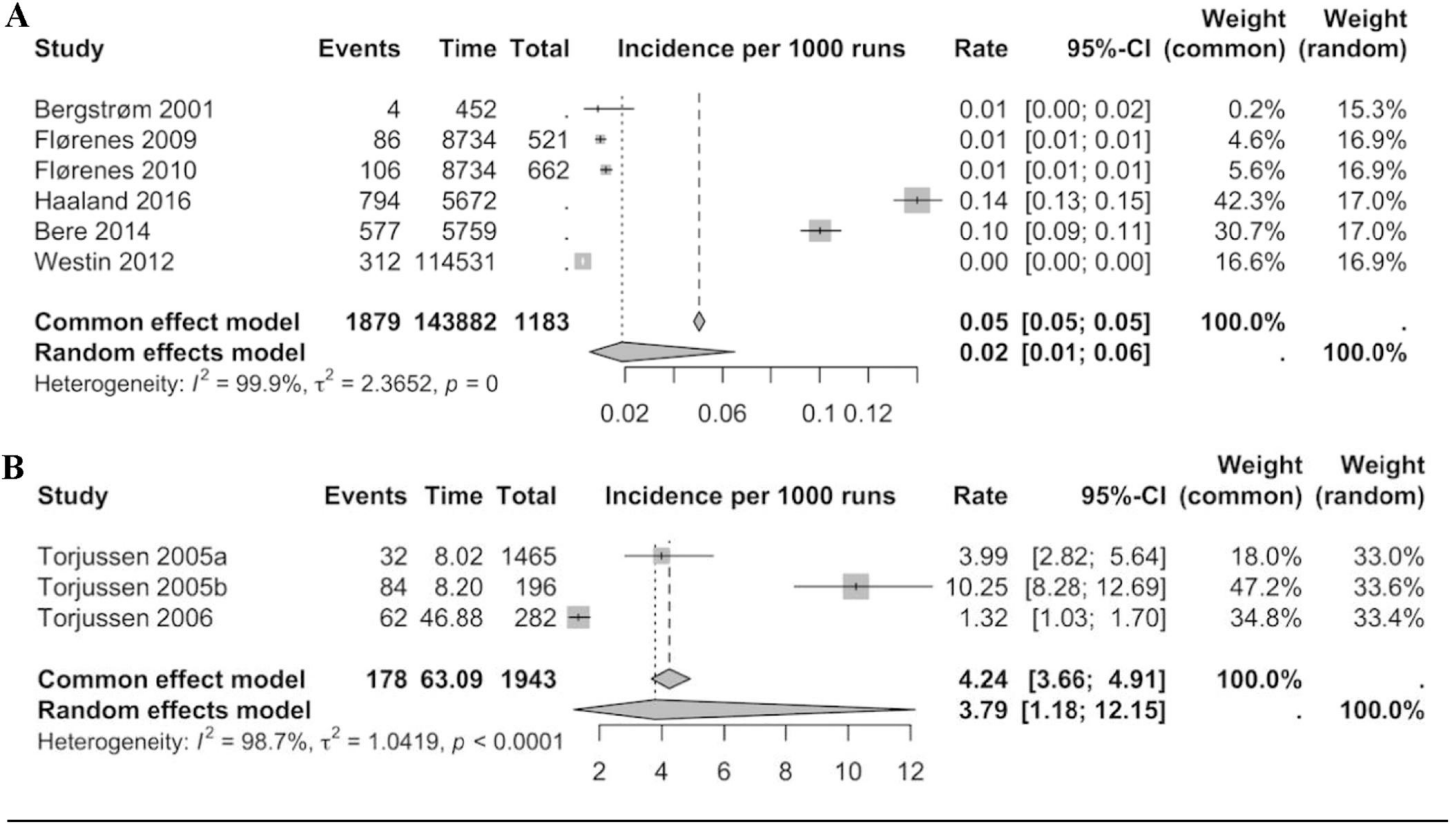
A. A forest plot illustrating the overall pooled injury incidence per 1000 runs in professional level alpine skiing, with exposures represented in thousands. B. A forest plot illustrating the overall pooled injury incidence per 1000 runs in professional level snowboarding, with exposures represented in thousands.

When stratified by sport, six studies focused on professional alpine skiing and reported 1879 injuries during 143,882,000 runs, yielding an incidence rate of 0.02 per 1000 runs (95% CI: 0.01–0.06, *I* ^*2*^ = *99.9%, τ^2^ = 2.3652*; number of studies = 6) (Figure 3A). In comparison, three studies on professional snowboarding recorded 178 injuries over just 63,090 runs, resulting in an injury rate of 3.8 per 1000 runs (95% CI: 1.2–12.2, *I* ^*2*^ = *98.7%, τ^2^=1.0419*; number of studies = 3) (Figure 3B).

### 3.3 Injury Incidences at the Amateur Level

**Figure 4:**
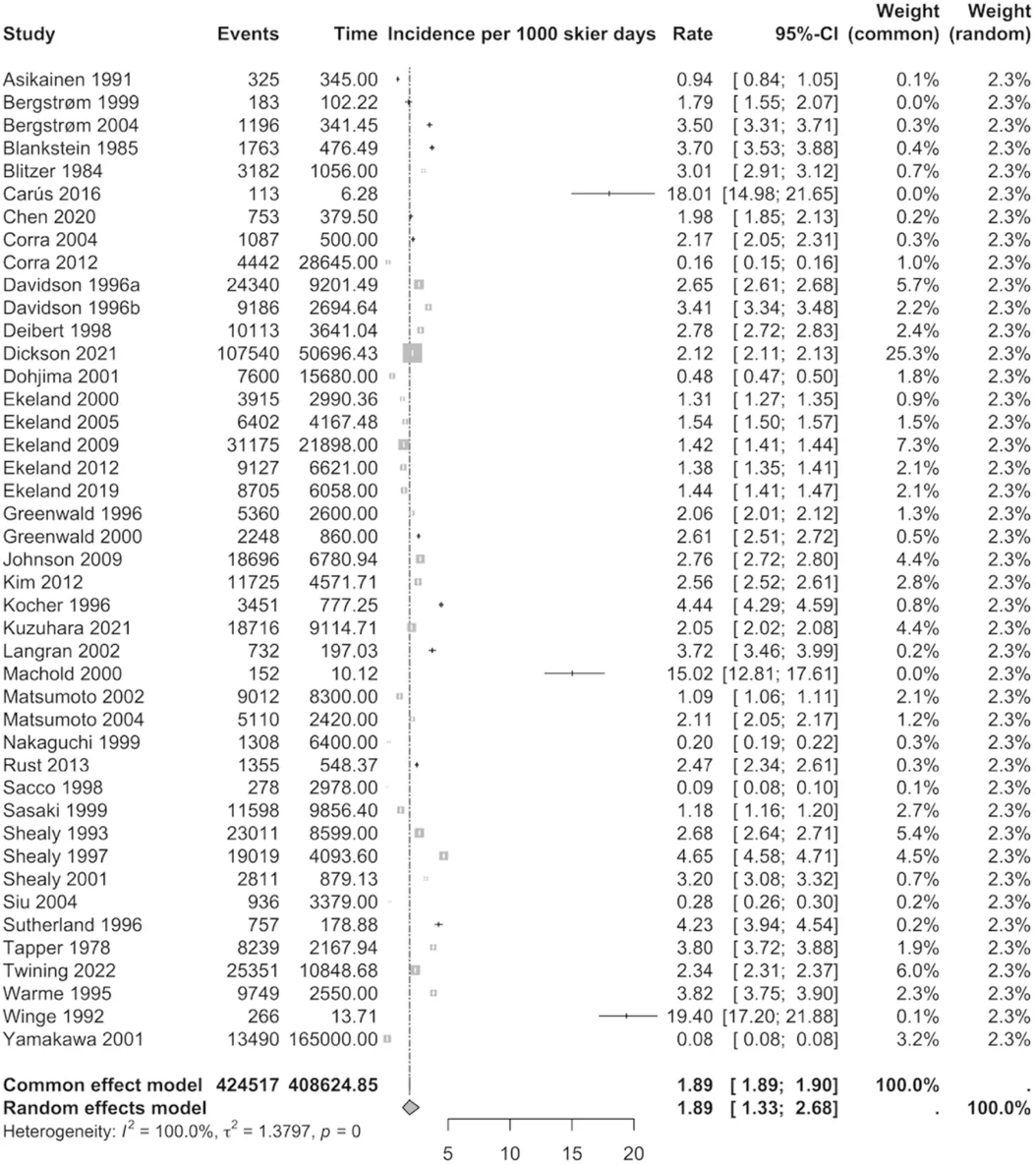
A forest plot illustrating the overall pooled injury incidence per 1000 skier‐days in amateur level alpine sports, with exposures represented in thousands.

Injury incidence among amateur alpine snow sports participants was reported in 43 studies, documenting a total of 424,517 injuries over 408,624,850 skier‐days. This corresponds to a pooled injury incidence of 1.9 injuries per 1000 skier‐days (95% CI: 1.3–2.7, *I* ^*2*^ = *100%, τ^2^ = 1.3797*; number of studies = 43) (Figure 4). A time‐stratified analysis, using the year 2000 as the division point, showed that 24 studies conducted before 2000 reported 150,050 injuries over 78,492,740 skier‐days, with an incidence of 2.3 per 1000 skier‐days (95% CI: 1.4–3.6, *I* ^*2*^ = *100%, τ^2^=1.3391*; number of studies = 24) (Supporting Information Appendix S5), whereas 16 studies conducted from 2000 onwards reported 251,233 injuries over 156,635,080 skier‐days, corresponding to an incidence of 1.7 per 1000 skier‐days (95% CI: 1.0–2.8, *I* ^*2*^ = *100%, τ^2^ = 1.0319*; number of studies = 16) (Supporting Information Appendix S6).

**Figure 5:**
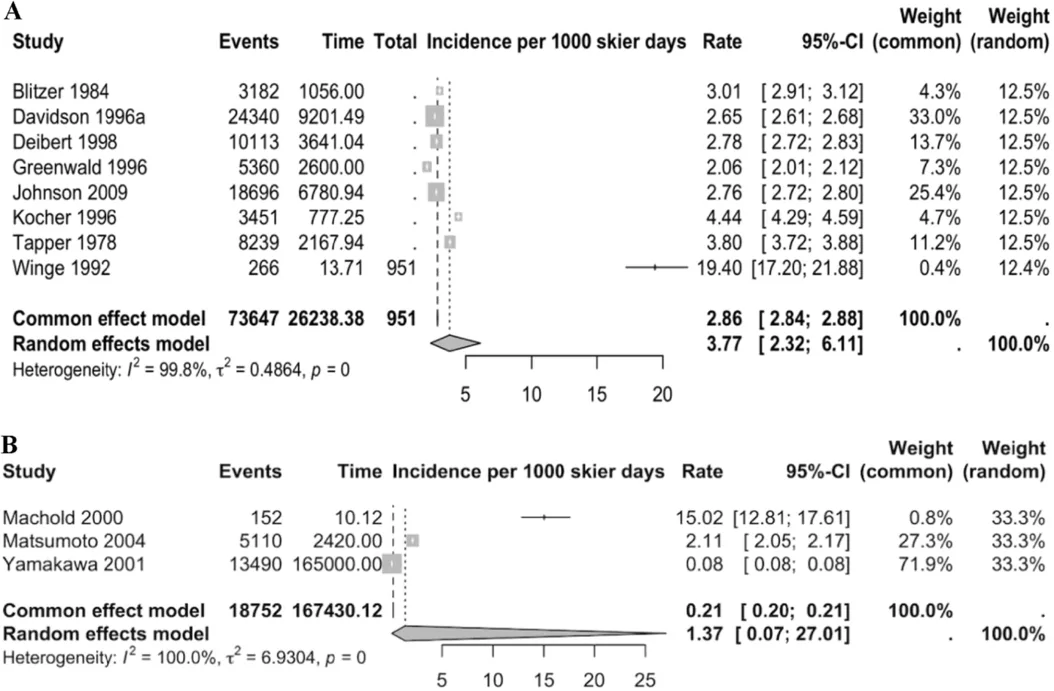
A. A forest plot illustrating the overall pooled injury incidence per 1000 skier‐days in amateur level alpine skiing, with exposures represented in thousands. B. A forest plot illustrating the overall pooled injury incidence per 1000 skier‐days in amateur level snowboarding, with exposures represented in thousands.

Specifically, eight studies focused solely on amateur alpine skiing, reporting 73,647 injuries across 26,238,380 skier‐days, resulting in an injury incidence of 3.8 per 1000 skier‐days (95% CI: 2.3–6.1, *I* ^*2*^ = *99.8%, τ^2^ = 0.4864*; number of studies = 8) (Figure 5A).

In comparison, three studies reported amateur snowboarding injuries, with 18,752 injuries occurring over 167,430,120 skier‐days, equating to an incidence of 1.4 per 1000 skier‐days (95% CI: 0.1–27.0, *I* ^*2*^ = *100%, τ^2^ = 6.9304*; number of studies = 3) (Figure 5B). The estimate should be interpreted cautiously because it was based on only three studies and had a wide confidence interval.

### 3.4 Injury Location

**Figure 6:**
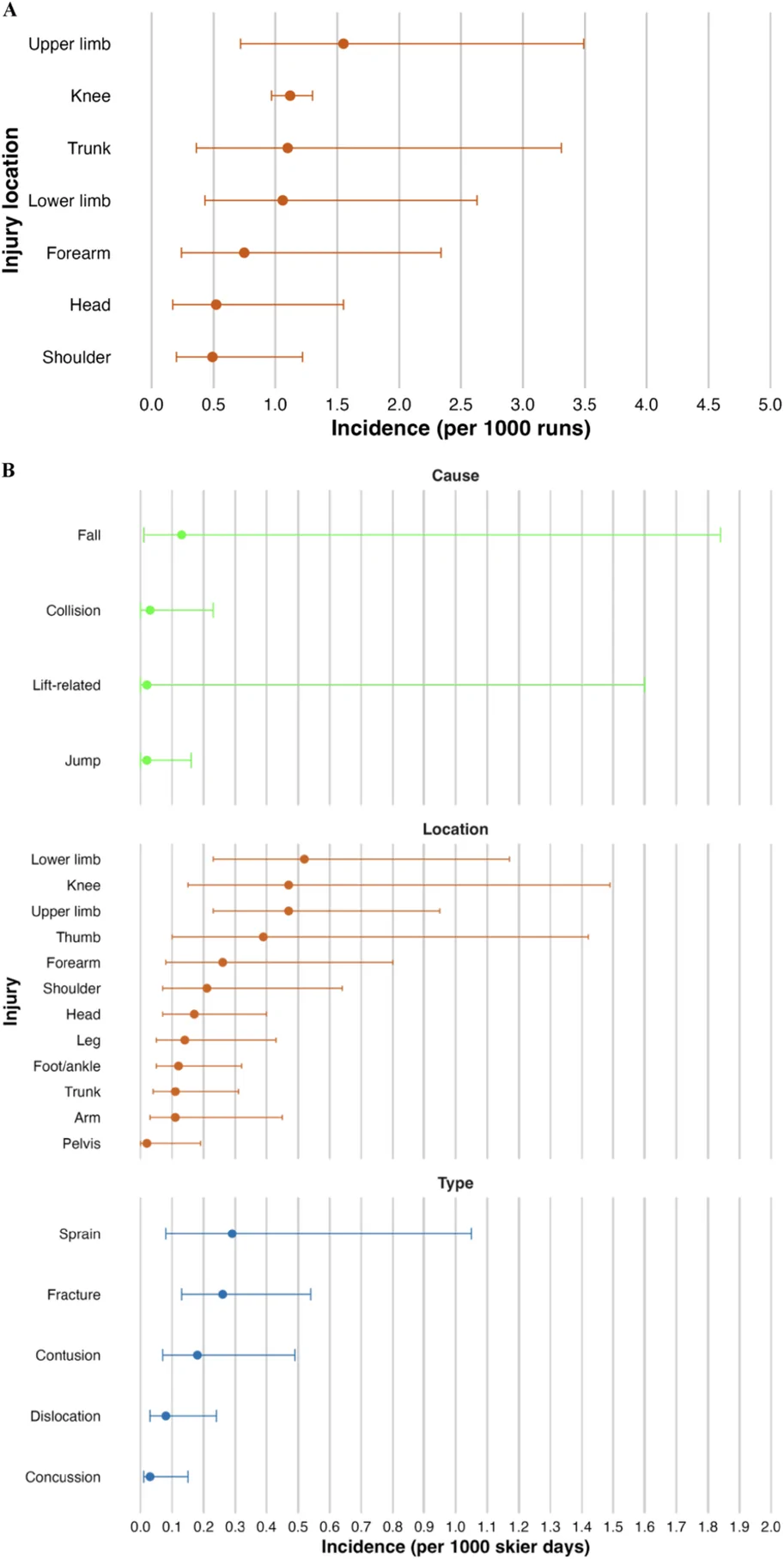
A. Injury incidence in professional level alpine sports, categorized by injury location. B. Injury incidence in amateur level alpine sports, categorized by injury location, injury type and injury cause.

At the professional level in alpine snow sports, upper limb injuries were the most frequently reported, followed by trunk and lower limb injuries. Among upper limb injuries, shoulder and forearm injuries were represented. Head injuries were less common but still notable. Knee injuries, though reported separately, also contributed to the overall injury burden. A detailed breakdown of injury sites and corresponding incidence rates is provided in Figure 6A and Supporting Information Appendix S7–13.

At the amateur level in alpine snow sports, lower limb, and upper limb injuries were the most frequently reported. Among upper limb injuries, thumb injuries were most common, while shoulder and arm injuries occurred less frequently. Lower limb injuries were also prevalent, particularly knee injuries, which had one of the highest reported incidences. Trunk, pelvis/hip, and foot/ankle injuries were less commonly reported. A comprehensive summary of injury sites and their incidence rates is provided in Figure 6B and Supporting Information Appendix S14–25.

### 3.5 Injury Type

At the amateur level in alpine snow sports, fractures and sprains were the most commonly reported injury types, both with the highest incidence rates. These were followed by contusions and dislocations, which occurred less frequently. Concussions were the least commonly reported injury type. A comprehensive summary of injury types and their incidence rates is provided in Figure 6B and Supporting Information Appendix S26–30. Three professional‐level studies [21, 40, 72] reported injury types; however, differences in exposure measures prevented meaningful pooling or direct quantitative comparison.

### 3.6 Injury Cause

At the amateur level in alpine snow sports, falls were the most commonly reported mechanism of injury, with the highest incidence rate among the studies reviewed. Collisions were the next most frequent. Lift‐related injuries and jumps were the least commonly reported. A detailed summary of injury mechanisms and their incidence rates is provided in Figure 6B and Supporting Information Appendix S31–34.

## 4 Discussion

This systematic review found that injury incidence in alpine snow sports is higher among professional snowboarders than among alpine skiers. In contrast, amateur participants exhibited markedly lower injury rates in snowboarding compared with alpine skiing. Because the exposure metrics differed between professional and amateur populations, direct comparisons between these groups could not be performed. These findings highlight how competition level, exposure type, and sport‐specific demands shape injury risk in alpine skiing and snowboarding.

Injury patterns differed between levels of participation. Among professionals, upper limb injuries were most common followed by trunk injuries, while amateurs most frequently sustained limb injuries, particularly to the knee. This variation likely reflects differences in performance demands and injury mechanisms: elite athletes are exposed to greater speeds, steeper slopes, more complex aerial maneuvers, and higher collision forces, whereas amateur injuries often stem from falls, inadequate technique, and less specialized conditioning. Our finding that fractures and sprains were the most frequent injury types at the amateur level aligns with earlier reports. For instance, a Danish study of recreational alpine skiers found that sprains (particularly to the knee and finger) were the most common injuries, followed by fractures and contusions [102]. Similarly, an Italian study reported that lower limb injuries accounted for 55.6% of injuries, followed by upper limb injuries (24.7%) and trunk injuries (11.4%); ligament and joint injuries were the most frequent injury type (36.8%), followed by fractures (35.7%) [103]. When injury incidence was analyzed in amateur alpine sports stratified by time period, rates were higher before the year 2000 compared to after, coinciding with the introduction of carving skis. However, this temporal difference cannot be attributed to changes in ski design alone and may also reflect other evolving factors, including changes in injury reporting practices, resort safety measures, helmet use, and case ascertainment.

The higher incidence of trunk injuries among professionals may be partly explained by biomechanical loading during high‐speed impacts and jumps, as well as more frequent exposure to rotational forces and direct collisions [104]. Protective equipment use, such as back protectors, has increased in recent years, but evidence of its effectiveness in elite competition remains mixed [10, 105]. Conversely, the predominance of knee injuries among amateurs may be associated with binding release settings, equipment fit, and reduced neuromuscular control, factors previously identified as modifiable through targeted interventions [106, 107]. A recent single‐center study of skiers and snowboarders undergoing ACL reconstruction further highlights the clinical relevance of knee injuries. In this cohort of 34 patients, only 47.1% returned to their pre‐injury sport, with younger age and shorter return‐to‐work time associated with successful return to sport [108]. These findings underscore the potential long‐term consequences of ACL injuries in alpine snow sports and the importance of effective injury prevention strategies.

At the amateur level, injury incidence was lower in studies published after 2000 than in those published before 2000. This temporal change coincided with the introduction of carving skis and increasing use of protective equipment, including helmets [8, 109]. However, this ecological comparison cannot establish that these developments caused the observed decline, as other factors may also have contributed, including changes in reporting practices, resort safety, case ascertainment, and course design. In alpine ski racing, modifications to course design and safety‐net systems have also been investigated as strategies to reduce injury risk by controlling speed and improving energy absorption [110]. These developments should therefore be considered when interpreting changes in injury incidence across decades.

To our knowledge, this is the largest systematic review and the first to provide pooled injury incidence estimates across all participation levels in alpine skiing and snowboarding. In contrast, previous reviews have largely focused exclusively on professional‐level athletes or specific disciplines whereas our review includes also amateur level skiers and snowboarders. The most recent systematic review and meta‐analysis examined only alpine skiing injuries and searched exclusively in PubMed; moreover, it did not report pooled injury incidence rates as presented in our review [12].

A systematic review and meta‐analysis of four studies on Olympic‐level alpine sports reported substantially lower injury rates than those observed in our pooled estimates for professional athletes. During four Winter Olympic Games, injury incidence was 17.4 for snowboarding (CI: 12.2–22.6; 171 injuries among 977 athletes), and 17.2 for alpine skiing (CI: 11.4–20.3; 217 injuries among 1250 athletes) [13]. These estimates are not directly comparable with our pooled rates of 0.02 per 1000 runs for professional alpine skiing and 3.8 per 1000 runs for professional snowboarding, because the studies used different exposure denominators. Moreover, the wide confidence intervals of our review likely reflect variability between included studies.

A previous narrative review of alpine skiing injuries, which included studies mainly from before and around the early 2000s, identified the lower extremity, particularly the knee, as the most commonly injured site [11]. This is consistent with our findings at the amateur level, where lower limb injuries, especially to the knee, predominated.

Another systematic review focusing on professional‐level skiing and snowboarding, including studies mostly from 2010 to 2020, reported an overall injury incidence of 3.49 per 1000 athlete‐days (CI: 2.97–4.01), with the lower extremity having the highest injury rate at 1.54 (CI: 1.24–1.84). Sport‐specific rates were 3.99 per 1000 athlete‐days for snowboarding (CI: 2.86–5.12) and 3.57 for alpine skiing (CI: 2.77–4.36) [14]. In comparison, our pooled estimates for professional snowboarding (3.8 per 1000 runs) and alpine skiing (1.4 per 1000 skier‐days), which may reflect differences in injury definitions, reporting thresholds, and study settings, particularly as our dataset included high‐risk events outside the Olympic context, where exposure to jumps, speed, and technical difficulty may be greater.

This review has several key strengths. It largely adhered to a predefined protocol, with the only deviation being the addition of a time‐stratified analysis. We consider this a strength rather than a limitation, as it provided valuable insights into the potential impact of major equipment changes on injury patterns, while still maintaining methodological rigor. To our knowledge, it is the first and largest review to provide pooled injury incidence estimates across both amateur and professional levels of alpine skiing and snowboarding. By categorizing injuries by type, anatomical location, and cause, it offers a comprehensive overview of injury patterns. The use of the Joanna Briggs Institute tool for risk of bias assessment further supports the quality of included studies. Lastly, the broad geographic distribution of studies enhances the global relevance and applicability of the findings.

This systematic review has several limitations. A primary concern is the variation in injury definitions across included studies, which may affect the accuracy of pooled incidence estimates. For instance, aggregating injuries without anatomical stratification could lead to misleading conclusions due to the substantial variation in injury rates by body region. To mitigate this, stratified analyzes were performed. Additionally, the predominance of studies focusing on amateur athletes limits the applicability of findings to professional populations. We did not have specific exposure units split by discipline (i.e., skiing vs. snowboarding) in all included studies. Therefore, we could not directly calculate discipline‐specific injury incidence across the total exposed population. Another key limitation is the high heterogeneity observed across studies, reflected in elevated I^2^ values and visual variability in forest plots, which suggests underlying differences in study design, populations, or reporting methods. This limits the interpretability of a single pooled estimate. The pooled injury estimates may be influenced by individual studies, as differences in study weight between fixed‐effects and random‐effects models can affect the overall results. Additionally, the absence of sex and age‐specific data in many original studies prevented analysis of sex and age differences. Another limitation is that time‐stratified analyzes could only be conducted at the amateur level, as the original studies did not provide sufficient information to stratify professional participants or to separate skiing and snowboarding. In addition, many studies lacked separate reporting for adult and youth participants, which restricted age‐based comparisons and limited the applicability of findings across different age groups. Not screening the reference lists of included studies, contacting original study authors, or searching the gray literature may have resulted in relevant studies being missed. In addition, restricting the search to English‐language studies may have limited the completeness and generalizability of the evidence identified. Finally, the certainty of evidence was not assessed using the GRADE approach, as it is not specifically designed for prevalence or incidence studies, which represents a methodological limitation. The median publication year of included studies was 2005, indicating that most data are relatively old; given substantial changes in safety measures, equipment technology, and competition formats since then, the reported injury patterns may not fully reflect the current situation.

From a prevention standpoint, these findings highlight the need for level‐ and sport‐specific injury prevention strategies. For professionals, strategies addressing trunk injuries may include appropriate use of back protectors, collision avoidance, and safe landing techniques, although evidence for the effectiveness of these measures in elite competition remains limited. For amateurs, appropriate binding‐release settings, equipment adjustment, neuromuscular training, falling techniques, and progressive skill development may help reduce lower‐limb injuries. Given that falls were the most common injury mechanism at the amateur level, slope design and hazard management may also contribute to injury prevention.

## 5 Conclusion

Injury incidence in alpine snow sports was higher among professional snowboarders than among alpine skiers. In contrast, amateur participants showed markedly lower injury rates in snowboarding compared with alpine skiing. Limb injuries were the most common in both groups, while trunk injuries were more frequent among professionals, likely due to high‐impact crashes and rotational forces. However, these comparisons should be interpreted cautiously because of heterogeneity and differences in study populations, injury definitions, reporting methods, and outcome measures. Most available data are outdated, with a median publication year of 2005. Since then, advances in equipment, such as carving skis, rocker profiles, and improved bindings, have increased speeds and altered biomechanical loads, potentially changing injury patterns. Contemporary, standardized studies, particularly among professional athletes, are needed to guide targeted injury‐prevention strategies and determine whether modern ski designs have altered the anatomical distribution and mechanisms of injuries in alpine snow sports.

## Author Contributions

**Juho Laaksonen:** conceptualization, writing – original draft, methodology, data curation, software. **Matias Vaajala:** writing – review and editing, methodology, software, data curation, formal analysis. **Rasmus Liukkonen:** methodology, software, data curation, writing – review and editing, conceptualization. **Oskari Pakarinen:** writing – review and editing, methodology, software, data curation. **Ville M Mattila:** methodology, writing – review and editing, supervision. **Ilari Kuitunen:** conceptualization, methodology, data curation, software, supervision, writing – review and editing.

## Funding

The authors have nothing to report.

## Ethics Statement

The authors have nothing to report.

## Conflicts of Interest

The authors declare no conflicts of interest.

## Transparency Statement

Corresponding author (Juho Laaksonen) affirms that this article is an honest, accurate, and transparent account of the study being reported; that no important aspects of the study have been omitted; and that any discrepancies from the study as planned (and, if relevant, registered) have been explained.

## Supporting information


Supporting File 1

Supporting File 2

Supporting File 3

Supporting File 4

## Data Availability

The data that supports the findings of this study are available in the supporting material of this article. All data extracted from the included studies are available in the supporting information. Our protocol was registered in the Prospero database CRD420251010125.
