# Supplementary material for: Epidemiology of Alpine Skiing and Snowboarding Injuries: A Systematic Review and Meta‐Analysis
Source: Health Sci Rep. 2026 Sep 13;9(9):e73252. doi: 10.1002/hsr2.73252 (PMC13572852; doi:10.1002/hsr2.73252)
Supplement: Supplementary file 2 [file HSR2-9-e73252-s003.docx]

**SEARCH STRATEGY**

**Search date:** March 14th 2025

**Filters:** None

**Time restrictions:** None

**Pubmed:** ((("alpine"[All Fields] OR "alpines"[All Fields]) AND ("skiing"[MeSH Terms] OR "skiing"[All Fields])) OR "slalom"[All Fields] OR ("skiing"[MeSH Terms] OR "skiing"[All Fields]) OR ("skiing"[MeSH Terms] OR "skiing"[All Fields] OR "snowboarding"[All Fields] OR "snowboard"[All Fields] OR "snowboarder"[All Fields] OR "snowboarder s"[All Fields] OR "snowboarders"[All Fields] OR "snowboards"[All Fields])) AND ("injurie"[All Fields] OR "injuried"[All Fields] OR "injuries"[MeSH Subheading] OR "injuries"[All Fields] OR "wounds and injuries"[MeSH Terms] OR ("wounds"[All Fields] AND "injuries"[All Fields]) OR "wounds and injuries"[All Fields] OR "injurious"[All Fields] OR "injury s"[All Fields] OR "injuryed"[All Fields] OR "injurys"[All Fields] OR "injury"[All Fields] OR ("fractur"[All Fields] OR "fractural"[All Fields] OR "fracture s"[All Fields] OR "fractures, bone"[MeSH Terms] OR ("fractures"[All Fields] AND "bone"[All Fields]) OR "bone fractures"[All Fields] OR "fracture"[All Fields] OR "fractured"[All Fields] OR "fractures"[All Fields] OR "fracturing"[All Fields]))

**Scopus:** ALL((alpine skiing OR slalom OR skiing OR snowboarding) AND (injury OR fracture))

**CINAHL:** (alpine skiing OR slalom OR skiing OR snowboarding) AND (injury OR fracture)

**Web of Science:** (((((TS = alpine skiing) OR (TS = slalom)) OR (TS = skiing)) OR (TS = snowboarding)) AND ((TS = injury) OR (TS = fracture)))
