# Supplementary material for: Epidemiology of Alpine Skiing and Snowboarding Injuries: A Systematic Review and Meta‐Analysis
Source: Health Sci Rep. 2026 Sep 13;9(9):e73252. doi: 10.1002/hsr2.73252 (PMC13572852; doi:10.1002/hsr2.73252)
Supplement: Supplementary file 3 [file HSR2-9-e73252-s004.docx]

**Appendix: Forest plots illustrating the anatomical locations of injuries and the types of injuries sustained in alpine sports, along with a table summarizing study characteristics and risk of bias**

| **Study** | **Equipment** | **Sex** | **Age** | **Level** | **Country** | **Study Period** | **Study design** | **Injury Definition** | **Injury situation** | **Injury type** |
| --- | --- | --- | --- | --- | --- | --- | --- | --- | --- | --- |
| Alhammoud 2020 | Skis | Both | Adult | Professional | France | 2013-2018 | Prospective study | Time loss | Competition & Training | All injury types |
| Asikainen 1991 | Not mentioned | Both | Both | Amateur | Finland | 1987-1990 | Prospective study | Medical attention | Leisure | All injury types |
| Barth 2020 | Skis | Both | Adult | Professional | Austria | 1997-2019 | Retrospective | Medical attention | Competition & Training | Severe injury events |
| Barth 2022 | Skis | Both | Both | Professional | Austria | 1997-2020 | Prospective study | Medical attention | Competition & Training | Severe injury events |
| Bere 2014 | Skis | Both | Adult | Professional | International | 2006-2012 | Retrospective | Medical attention/time loss | Competition & Training | All injury types |
| Bergstrøm 1999 | Both | Both | Both | Amateur | Norway | 1990-1992 | Prospective study | Medical attention | Leisure | All injury types |
| Bergstrøm 2001 | Skis | Both | Youth | Professional | Norway | Not reported | Prospective study | Medical attention | Competition & Training | All injury types |
| Bergstrøm 2004 | Both | Both | Both | Amateur | Norway | 1990-1996 | Prospective study | Medical attention | Leisure | All injury types |
| Blankstein 1985 | Not mentioned | Both | Both | Amateur | Switzerland | 1976-1982 | Prospective study | Medical attention | Leisure | All injury types |
| Blitzer 1984 | Skis | Both | Both | Amateur | Sweden | 1972-1973 | Prospective study | Medical attention | Leisure | All injury types |
| Carús 2016 | Both | Both | Both | Amateur | Spain | 2013-2014 | Retrospective | Medical attention | Leisure | All injury types |
| Chen 2020 | Both | Both | Both | Amateur | China | 2018-2019 | Retrospective | Medical attention | Leisure | All injury types |
| Corra 2004 | Both | Both | Both | Amateur | Italy | 2001-2002 | Prospective study | Medical attention | Leisure | All injury types |
| Corra 2012 | Both | Both | Both | Amateur | Italy | 2001-2005 | Retrospective | Medical attention | Leisure | All injury types |
| Davidson 1996a | Skis | Both | Both | Amateur | USA | 1983-1992 | Retrospective | Medical attention | Leisure | All injury types |
| Davidson 1996b | Both | Both | Both | Amateur | USA | 1989-1993 | Retrospective | Medical attention | Leisure | All injury types |
| Deibert 1998 | Skis | Both | Both | Amateur | France | 1972-1981 | Prospective study | Medical attention | Leisure | All injury types |
| Dickson 2021 | Both | Both | Both | Amateur | Canada | 2008-2018 | Prospective study | Medical attention | Leisure | All injury types |
| Dohjima 2001 | Both | Both | Youth | Amateur | Japan | 1988-1997 | Prospective study | Medical attention | Leisure | All injury types |
| Ekeland 1985 | Skis | Both | Both | Professional | Norway | 1981-1982 | Prospective study | Not mentioned | Competition & Training | All injury types |
| Ekeland 2000 | Both | Both | Both | Amateur | Norway | 1996-1998 | Prospective study | Medical attention | Leisure | All injury types |
| Ekeland 2005 | Both | Both | Both | Amateur | Norway | 2000-2002 | Prospective study | Medical attention | Leisure | All injury types |
| Ekeland 2009 | Both | Both | Both | Amateur | Norway | 1996-2006 | Prospective study | Medical attention | Leisure | All injury types |
| Ekeland 2012 | Both | Both | Both | Amateur | Norway | 2008-2010 | Prospective study | Medical attention | Leisure | All injury types |
| Ekeland 2019 | Both | Both | Both | Amateur | Norway | 2010-2012 | Retrospective | Medical attention | Leisure | All injury types |
| Flørenes 2009 | Skis | Both | Adult | Professional | International | 2006-2008 | Retrospective | Medical attention | Competition & Training | All injury types |
| Flørenes 2010 | Skis | Both | Adult | Professional | International | 2006-2009 | Retrospective | Medical attention | Competition & Training | All injury types |
| Fukuda 2001 | Both | Both | Both | Amateur | Japan | 1994-1999 | Prospective study | Head-injury | Leisure | Head injuries |
| Gallo-Vallejo 2017 | Both | Both | Adult | semi-professional | Spain | 2015 | Prospective study | Medical attention | kisajatraining | All injury types |
| Gao 2024 | lauta | Both | Both | Amateur | China | 2018-2019 | Prospective study | Medical attention | Leisure | All injury types |
| Gertzbein 2012 | Both | Both | Both | Amateur | USA | 2005-2010 | Retrospective | Medical attention | Leisure | Spine fracture |
| Gil 2017 | Both | Both | Both | Amateur | USA | 2010-2014 | Retrospective | Medical attention | Leisure | Concussion |
| Graves 2013 | Both | Both | Youth | Amateur | USA | 1996-2010 | Retrospective | Clinical diagnosis | Leisure | Head injuries |
| Greenwald 1996 | Skis | Both | Both | Amateur | USA | 1989-1993 | Retrospective | Medical attention | Leisure | All injury types |
| Greenwald 2000 | Both | Both | Both | Amateur | USA | 1998-1999 | Prospective study | Medical attention | Leisure | All injury types |
| Haaland 2016 | Skis | Both | Adult | Professional | Norway | 2016-2015 | Retrospective | Time loss | Competition & Training | All injury types |
| Hentschel 2001 | Snowboard | Both | Both | Amateur | Canada | 1992-1997 | Retrospective | Medical attention | Leisure | Head injuries |
| Hosaka 2020 | Snowboard | Both | Both | Amateur | Japan | 2006-2017 | Prospective study | Medical attention | Leisure | Spinal injuries |
| Ishimaru 2011 | Snowboard | Both | Both | Amateur | Japan | 2004-2009 | Retrospective | Medical attention | Leisure | Lower extremity injury |
| Johansen 2015 | Skis | Both | Adult | Professional | Norway | 2008-2013 | Retrospective | Medical attention | Competition & Training | All injury types |
| Johnson 2009 | Skis | Both | Both | Amateur | Usa | 1972-2006 | Prospective study | Medical attention | Leisure | All injury types |
| Kim 2012 | Both | Both | Both | Amateur | USA | 1988-2006 | Prospective study | Medical attention | Leisure | All injury types |
| Kocher 1996 | Skis | Both | Both | Amateur | USA | 1990-1993 | Retrospective | Medical attention | Leisure | All injury types |
| Kuzuhara 2021 | Both | Both | Both | Amateur | Japan | 2012-2018 | Prospective study | Medical attention | Leisure | All injury types |
| Langran 2002 | Both | Both | Both | Amateur | Scotland | 1999-2000 | Prospective study | Medical attention | Leisure | All injury types |
| Laporte 2000 | Both | Both | Both | Amateur | France | 1992-1999 | Prospective study | Medical attention | Leisure | ACL injuries |
| Lindman 2024 | Skis | Both | Youth | Amateur | Sweden | 2013-2018 | Prospective study | Medical attention | Leisure | All injury types |
| Machold 2000 | Snowboard | Both | Youth | Amateur | Austria | 1996-1997 | Prospective study | All injuries | Leisure | All injury types |
| Machold 2002 | Snowboard | Both | Youth | Amateur | Austria | Not reported | RCT | Medical attention | Leisure | Wrist injuries |
| Matsumoto 2002 | Both | Both | Both | Amateur | Japan | 1995-2000 | Prospective study | Medical attention | Leisure | All injury types |
| Matsumoto 2004 | Snowboard | Both | Both | Amateur | Japan | 1998-2001 | Prospective study | Medical attention | Leisure | All injury types |
| Müller 2017 | Skis | Both | Youth | Professional | Austria | 2014-2016 | Prospective study | Time loss | Competition & Training | All injury types |
| Nakaguchi 1999 | Both | Both | Both | Amateur | Japan | 1995-1997 | Prospective study | Medical attention | Leisure | All injury types |
| Oberle 2021 | Both | Both | Both | Amateur | USA | 2012-2017 | Retrospective | Medical attention | Leisure | Clavicle fracture |
| Ogawa 2010 | Snowboard | Both | Both | Amateur | Japan | 1998-2007 | Prospective study | Medical attention | Leisure | Hip fracture |
| Pujol 2007 | Skis | Both | Both | Professional | France | 1980-2005 | Prospective study | Medical attention | Competition & Training | ACL injuries |
| Quinlan 2020 | Both | Both | Both | Amateur | USA | 1972-2012 | Prospective study | Medical attention | Leisure | Wrist fracture |
| Rønning 2000 | Both | Both | Both | Amateur | Norway | 1997 | Prospective study | Medical attention | Leisure | All injury types |
| Ruedl 2012 | Snowboard | Both | Youth | Professional | Canada | 2012 | Prospective study | Medical attention | Competition & Training | All injury types |
| Rust 2013 | Both | Both | Both | Amateur | USA | 2008-2010 | Prospective study | Medical attention | Leisure | All injury types |
| Sacco 1998 | Both | Both | Both | Amateur | France | 1990-1995 | Retrospective | Medical attention | Leisure | All injury types |
| Sakamoto 2008 | Both | Both | Both | Amateur | Japan | 2000-2005 | Prospective study | Medical attention | Leisure | All injury types |
| Sasaki 1999 | Both | Both | Both | Amateur | Japan | 1990-1997 | Prospective study | Medical attention | Leisure | All injury types |
| SHEALY 1993 | Both | Both | Both | Amateur | USA | 1988-1990 | Retrospective | Not mentioned | Leisure | All injury types |
| Shealy 1997 | Both | Both | Both | Amateur | USA | 1988-1995 | Retrospective | Medical attention | Leisure | All injury types |
| Shealy 2001 | Skitboard | Both | Both | Amateur | USA | 1997-1999 | Retrospective | Not mentioned | Leisure | All injury types |
| Siu 2004 | Both | Both | Both | Amateur | Australia | 1994-2002 | Retrospective | Medical attention | Leisure | All injury types |
| Steenstrup 2011 | Both | Both | Adult | Professional | International | 2006-2010 | Retrospective | Time loss | Competition & Training | All injury types |
| Steenstrup 2014 | Both | Both | Adult | Professional | Norway | 2006-2013 | Retrospective | Medical attention | Competition & Training | Head injuries |
| Stenroos 2015 | Both | Both | Both | Amateur | Finland | 2006-2012 | Retrospective | Medical attention | Leisure | All injury types |
| Sutherland 1996 | Both | Both | Both | Amateur | Scotland | 1994-1995 | Prospective study | Medical attention | Leisure | All injury types |
| Tapper 1978 | Skis | Both | Both | Amateur | USA | 1952-1976 | Prospective questionnaire | Medical attention | Leisure | All injury types |
| Tarazi 1999 | Both | Both | Both | Amateur | Canada | 1994-1996 | Retrospective | Medical attention | Leisure | Spinal injuries |
| Torjussen 2005a | Snowboard | Both | Both | Professional | Norway | 2002 | Prospective study | Medical attention | Competition & Training | All injury types |
| Torjussen 2005b | Snowboard | Both | Both | Professional | Norway | 2002 | Retrospective | Medical attention | Competition & Training | All injury types |
| Torjussen 2006 | Snowboard | Both | Adult | Professional | Switzerland | 2002-2003 | Retrospective | Medical attention | Competition & Training | All injury types |
| Twining 2022 | Both | Both | Both | Amateur | USA | 1972-2018 | Prospective study | Medical attention | Leisure | All injury types |
| Viola 1999 | Skis | Both | Adult | Professional | USA | 1991-1992 | Prospective study | Medical attention | Competition & Training | ACL injuries |
| Warme 1995 | Both | Both | Both | Amateur | USA | 1982-1993 | Retrospective | Medical attention | Leisure | All injury types |
| Westin 2012 | Skis | Both | Youth | Professional | Sweden | 2006-2011 | Prospective study | Time loss | Competition & Training | All injury types |
| Winge 1992 | Skis | Both | Both | Amateur | Denmark | 1987 | Prospective study | Medical attention | Leisure | All injury types |
| Yamakawa 2001 | Snowboard | Both | Both | Amateur | Japan | 1988-2000 | Retrospective | Medical attention | Leisure | All injury types |

**Appendix 1. Characteristics of the included studies.**

| **Study** | **Was the sample frame appropriate to address the target population?** | **Were study participants sampled in an appropriate way?** | **Was the sample size adequate?** | **Were the study subjects and the setting described in detail?** | **Was the data analysis conducted with sufficient coverage of the identified sample?** | **Were valid methods used for the identification of the condition?** | **Was the condition measured in a standard, reliable way for all participants?** | **Was there appropriate statistical analysis?** | **Was the response rate adequate, and if not, was the low response rate managed appropriately?** | **Quality** |
| --- | --- | --- | --- | --- | --- | --- | --- | --- | --- | --- |
| Alhammoud 2020 | Yes | Yes | No | No | Yes | Yes | Yes | Yes | Yes | Medium |
| Asikainen 1991 | Yes | Yes | Yes | No | Yes | Yes | Yes | Yes | No | Medium |
| Barth 2020 | Yes | Yes | Yes | No | Yes | No | No | Yes | No | Low |
| Barth 2022 | Yes | Yes | Yes | No | Yes | No | No | Yes | No | Low |
| Bere 2014 | Yes | No | Yes | No | Yes | No | No | Yes | No | Low |
| Bergstrøm 1999 | Yes | Yes | Yes | No | Yes | Yes | Yes | Yes | No | High |
| Bergstrøm 2001 | Yes | No | Yes | No | Yes | No | No | Yes | No | Low |
| Bergstrøm 2004 | Yes | Yes | Yes | No | Yes | Yes | Yes | Yes | No | Medium |
| Blankstein 1985 | Yes | Yes | Yes | No | Yes | Yes | Yes | Yes | Yes | High |
| Blitzer 1984 | Yes | Yes | Yes | No | Yes | Yes | Yes | Yes | Yes | High |
| Carús 2016 | Yes | Yes | No | Yes | Yes | Yes | Yes | Yes | No | Medium |
| Chen 2020 | Yes | Yes | Yes | No | Yes | Yes | Yes | Yes | No | Medium |
| Corra 2004 | Yes | Yes | Yes | No | Yes | Yes | No | Yes | No | Medium |
| Corra 2012 | Yes | No | Yes | No | Yes | No | No | Yes | No | Low |
| Davidson 1996 | Yes | Yes | Yes | No | Yes | Yes | No | Yes | No | Medium |
| Davidson 1996 | Yes | Yes | Yes | No | Yes | Yes | No | Yes | No | Medium |
| Deibert 1998 | Yes | Yes | Yes | No | Yes | Yes | No | Yes | No | Medium |
| Dickson 2021 | Yes | Yes | Yes | Yes | Yes | No | No | Yes | No | Medium |
| Dohjima 2001 | Yes | No | Yes | No | Yes | No | No | Yes | Yes | Low |
| Ekeland 1985 | Yes | Yes | No | No | No | Yes | No | Yes | No | Low |
| Ekeland 2000 | Yes | Yes | Yes | No | Yes | Yes | No | Yes | Yes | Medium |
| Ekeland 2005 | Yes | Yes | Yes | No | Yes | Yes | No | Yes | Yes | Medium |
| Ekeland 2006 | Yes | Yes | Yes | No | Yes | Yes | No | Yes | Yes | Medium |
| Ekeland 2009 | Yes | Yes | Yes | No | Yes | No | No | Yes | Yes | Medium |
| Ekeland 2012 | Yes | Yes | Yes | No | Yes | Yes | No | Yes | Yes | Medium |
| Ekeland 2019 | Yes | Yes | Yes | No | Yes | No | No | Yes | No | Low |
| Flørenes 2009 | Yes | Yes | Yes | No | Yes | Yes | No | Yes | Yes | Medium |
| Flørenes 2010 | Yes | Yes | Yes | No | Yes | Yes | No | Yes | Yes | Medium |
| Fukuda 2001 | Yes | Yes | Yes | Yes | Yes | Yes | No | Yes | No | Medium |
| Gallo-Vallejo 2017 | Yes | Yes | No | No | Yes | Yes | Yes | Yes | No | Medium |
| Gao 2024 | Yes | Yes | Yes | No | Yes | Yes | Yes | Yes | Yes | High |
| Gertzbein 2012 | Yes | Yes | No | No | Yes | Yes | Yes | Yes | No | Medium |
| Gil 2017 | Yes | Yes | Yes | No | Yes | Yes | No | Yes | Yes | Medium |
| Graves 2013 | Yes | Yes | Yes | Yes | Yes | Yes | No | Yes | Yes | High |
| Greenwald 1996 | Yes | Yes | Yes | No | Yes | Yes | No | Yes | Yes | Medium |
| Greenwald 2000 | Yes | Yes | Yes | No | Yes | Yes | Yes | Yes | Yes | High |
| Haaland 2016 | Yes | Yes | Yes | No | Yes | No | No | Yes | Yes | Medium |
| Hentschel 2001 | Yes | Yes | No | No | Yes | Yes | Yes | Yes | Yes | Medium |
| Hosaka 2020 | Yes | Yes | No | Yes | Yes | No | No | Yes | Yes | Medium |
| Ishimaru 2011 | Yes | Yes | Yes | Yes | Yes | Yes | No | Yes | Yes | High |
| Johansen 2015 | Yes | Yes | No | No | Yes | Yes | No | Yes | Yes | Medium |
| Johnson 2009 | Yes | Yes | Yes | No | Yes | Yes | Yes | Yes | Yes | High |
| Kim 2012 | Yes | Yes | Yes | Yes | Yes | Yes | No | Yes | Yes | High |
| Kocher 1996 | Yes | Yes | Yes | No | Yes | Yes | No | Yes | Yes | Medium |
| Kuzuhara 2021 | Yes | Yes | Yes | No | Yes | Yes | No | Yes | Yes | Medium |
| Langran 2002 | Yes | Yes | Yes | No | Yes | No | No | Yes | Yes | Medium |
| Laporte 2000 | No | Yes | Yes | No | Yes | No | No | Yes | Yes | Low |
| Lindman 2024 | Yes | Yes | No | No | Yes | Yes | No | Yes | Yes | Medium |
| Machold 2000 | Yes | Yes | Yes | No | Yes | Yes | No | Yes | Yes | Medium |
| Machold 2002 | No | Yes | No | No | No | Yes | Yes | Yes | Yes | Low |
| Matsumoto 2002 | Yes | Yes | Yes | Yes | Yes | No | No | Yes | Yes | Medium |
| Matsumoto 2004 | Yes | Yes | Yes | Yes | Yes | No | No | Yes | Yes | Medium |
| Müller 2017 | Yes | Yes | No | Yes | Yes | Yes | No | Yes | Yes | Medium |
| Nakaguchi 1999 | Yes | Yes | Yes | No | Yes | Yes | No | Yes | No | Medium |
| Oberle 2021 | Yes | Yes | Yes | No | Yes | Yes | Yes | Yes | Yes | High |
| Ogawa 2010 | Yes | Yes | No | No | Yes | Yes | No | Yes | Yes | Medium |
| Pujol 2007 | Yes | Yes | Yes | No | Yes | Yes | Yes | Yes | Yes | High |
| Quinlan 2020 | Yes | Yes | Yes | Yes | Yes | Yes | Yes | Yes | Yes | High |
| Ruedl 2012 | Yes | Yes | No | No | Yes | Yes | Yes | Yes | Yes | Medium |
| Rust 2013 | Yes | Yes | Yes | Yes | Yes | Yes | No | Yes | Yes | High |
| Rønning 2000 | No | No | No | No | Yes | No | No | Yes | Yes | Low |
| Sacco 1998 | Yes | Yes | No | No | Yes | Yes | No | Yes | Yes | Medium |
| Sakamoto 2008 | Yes | Yes | Yes | No | Yes | Yes | Yes | Yes | Yes | High |
| Sasaki 1999 | Yes | Yes | Yes | No | Yes | Yes | No | Yes | Yes | Medium |
| SHEALY 1993 | Yes | Yes | Yes | Yes | Yes | Yes | No | Yes | Yes | High |
| Shealy 1997 | Yes | Yes | Yes | No | Yes | Yes | No | Yes | Yes | Medium |
| Shealy 2001 | Yes | Yes | No | No | No | No | No | Yes | No | Low |
| Siu 2004 | Yes | Yes | Yes | No | Yes | Yes | Yes | Yes | Yes | High |
| Steenstrup 2011 | Yes | Yes | Yes | No | Yes | Yes | No | Yes | Yes | Medium |
| Steenstrup 2014 | Yes | Yes | Yes | No | Yes | Yes | Yes | Yes | Yes | High |
| Stenroos 2015 | Yes | Yes | Yes | No | Yes | Yes | Yes | Yes | Yes | High |
| Sutherland 1996 | Yes | Yes | Yes | No | Yes | Yes | No | Yes | Yes | Medium |
| Tapper 1978 | Yes | Yes | Yes | No | Yes | Yes | No | Yes | Yes | Medium |
| Tarazi 1999 | Yes | Yes | Yes | No | Yes | Yes | No | Yes | Yes | Medium |
| Torjussen 2005 | Yes | Yes | Yes | Yes | Yes | Yes | No | Yes | Yes | High |
| Torjussen 2006 | Yes | Yes | Yes | Yes | Yes | Yes | Yes | Yes | Yes | High |
| Twining 2022 | Yes | Yes | Yes | Yes | Yes | Yes | No | Yes | Yes | High |
| Viola 1999 | Yes | Yes | Yes | Yes | Yes | Yes | No | Yes | Yes | High |
| Warme 1995 | Yes | Yes | Yes | No | Yes | No | No | Yes | Yes | Medium |
| Westin 2012 | Yes | Yes | Yes | No | Yes | No | No | Yes | Yes | Medium |
| Winge 1992 | Yes | Yes | Yes | No | Yes | No | No | Yes | Yes | Medium |
| Yamakawa 2001 | Yes | Yes | Yes | No | Yes | Yes | No | Yes | No | Medium |

**Appendix 2. The risk of bias evaluated using the Joanna Briggs Institute Critical Appraisal Checklist for Prevalence Studies**

**Appendix 3. A forest plot illustrating the overall pooled injury incidence per 1 skier seasons in professional level alpine sports, with exposures represented in thousands.**

**Appendix 4. A forest plot illustrating the overall pooled injury incidence per 1,000 hours in professional level alpine sports, with exposures represented in thousands.**

**Appendix 5. A forest plot illustrating the overall pooled injury incidence per 1,000 skier days in amateur level alpine sports before year 2000, with exposures represented in thousands.**

**Appendix 6. A forest plot illustrating the overall pooled injury incidence per 1,000 skier days in amateur level alpine sports after year 2000, with exposures represented in thousands.**

**Appendix 7. A forest plot showing the pooled incidence of head and neck injuries per 1,000 runs in professional level alpine sports, with exposures presented in thousands**

**Appendix 8. A forest plot showing the pooled incidence of upper limb injuries per 1,000 runs in professional level alpine sports, with exposures presented in thousands**

**Appendix 9. A forest plot showing the pooled incidence of shoulder injuries per 1,000 runs in professional level alpine sports, with exposures presented in thousands**

**Appendix 10. A forest plot showing the pooled incidence of forearm injuries per 1,000 runs in professional level alpine sports, with exposures presented in thousands**

**Appendix 11. A forest plot showing the pooled incidence of trunk injuries per 1,000 runs in professional level alpine sports, with exposures presented in thousands**

**Appendix 12. A forest plot showing the pooled incidence of lower limb injuries per 1,000 runs in professional level alpine sports, with exposures presented in thousands**

**Appendix 13. A forest plot showing the pooled incidence of knee injuries per 1,000 runs in professional level alpine sports, with exposures presented in thousands**

**Appendix 14. A forest plot showing the pooled incidence of head and neck injuries per 1,000 skier days in amateur level alpine sports, with exposures presented in thousands**

**Appendix 15. A forest plot showing the pooled incidence of upper limb injuries per 1,000 skier days in amateur level alpine sports, with exposures presented in thousands**

**Appendix 16. A forest plot showing the pooled incidence of shoulder injuries per 1,000 skier days in amateur level alpine sports, with exposures presented in thousands**

**Appendix 17. A forest plot showing the pooled incidence of arm injuries per 1,000 skier days in amateur level alpine sports, with exposures presented in thousands**

**Appendix 18. A forest plot showing the pooled incidence of forearm injuries per 1,000 skier days in amateur level alpine sports, with exposures presented in thousands**

**Appendix 19. A forest plot showing the pooled incidence of thumb injuries per 1,000 skier days in amateur level alpine sports, with exposures presented in thousands**

**Appendix 20. A forest plot showing the pooled incidence of trunk injuries per 1,000 skier days in amateur level alpine sports, with exposures presented in thousands**

**Appendix 21. A forest plot showing the pooled incidence of lower limb injuries per 1,000 skier days in amateur level alpine sports, with exposures presented in thousands**

**Appendix 22. A forest plot showing the pooled incidence of pelvis injuries per 1,000 skier days in amateur level alpine sports, with exposures presented in thousands**

**Appendix 23. A forest plot showing the pooled incidence of knee injuries per 1,000 skier days in amateur level alpine sports, with exposures presented in thousands**

**Appendix 24. A forest plot showing the pooled incidence of leg injuries per 1,000 skier days in amateur level alpine sports, with exposures presented in thousands**

**Appendix 25. A forest plot showing the pooled incidence of foot/ankle injuries per 1,000 skier days in amateur level alpine sports, with exposures presented in thousands**

**Appendix 26. A forest plot showing the pooled incidence of concussions per 1,000 skier days in amateur level alpine sports, with exposures presented in thousands**

**Appendix 27. A forest plot showing the pooled incidence of sprains per 1,000 skier days in amateur level alpine sports, with exposures presented in thousands**

**Appendix 28. A forest plot showing the pooled incidence of contusions per 1,000 skier days in amateur level alpine sports, with exposures presented in thousands**

**Appendix 29. A forest plot showing the pooled incidence of fractures per 1,000 skier days in amateur level alpine sports, with exposures presented in thousands**

**Appendix 30. A forest plot showing the pooled incidence of dislocations per 1,000 skier days in amateur level alpine sports, with exposures presented in thousands**

**Appendix 31. A forest plot presenting the pooled incidence of injuries per 1,000 skier-days caused by collisions in amateur-level alpine snow sports is shown, with exposure values reported in thousands**

**Appendix 32. A forest plot presenting the pooled incidence of injuries per 1,000 skier-days caused by jumps in amateur-level alpine snow sports is shown, with exposure values reported in thousands**

**Appendix 33. A forest plot presenting the pooled incidence of injuries per 1,000 skier-days caused by falls in amateur-level alpine snow sports is shown, with exposure values reported in thousands**

**Appendix 34. A forest plot presenting the pooled incidence of injuries per 1,000 skier-days caused by lift related issues in amateur-level alpine snow sports is shown, with exposure values reported in thousands**
